# Single nucleotide polymorphisms in the *MYLKP1* pseudogene are associated with increased colon cancer risk in African Americans

**DOI:** 10.1371/journal.pone.0200916

**Published:** 2018-08-30

**Authors:** Heather Lynn, Xiaoguang Sun, Djanybek Ayshiev, Jessica H. Siegler, Alicia N. Rizzo, Jason H. Karnes, Manuel Gonzales Garay, Ting Wang, Nancy Casanova, Sara M. Camp, Nathan A. Ellis, Joe GN Garcia

**Affiliations:** 1 Department of Medicine, University of Arizona Health Sciences, Tucson, AZ United States of America; 2 Graduate Interdisciplinary Program in Physiological Sciences, University of Arizona, Tucson, AZ United States of America; 3 Department of Medicine, University of Illinois at Chicago, Chicago, IL United States of America; 4 Department of Pharmacy Practice and Science, College of Pharmacy, University of Arizona, Tucson, AZ United States of America; 5 Department of Cell and Molecular Medicine, University of Arizona Health Sciences, Tucson, AZ United States of America; University of South Alabama Mitchell Cancer Institute, UNITED STATES

## Abstract

**Introduction:**

Pseudogenes are paralogues of functional genes historically viewed as defunct due to either the lack of regulatory elements or the presence of frameshift mutations. Recent evidence, however, suggests that pseudogenes may regulate gene expression, although the functional role of pseudogenes remains largely unknown. We previously reported that *MYLKP1*, the pseudogene of *MYLK* that encodes myosin light chain kinase (MLCK), is highly expressed in lung and colon cancer cell lines and tissues but not in normal lung or colon. The *MYLKP1* promoter is minimally active in normal bronchial epithelial cells but highly active in lung adenocarcinoma cells. In this study, we further validate *MYLKP1* as an oncogene via elucidation of the functional role of *MYLKP1* genetic variants in colon cancer risk.

**Methods:**

Proliferation and migration assays were performed in *MYLKP1-*transfected colon and lung cancer cell lines (H441, A549) and commercially-available normal lung and colon cells. Fourteen *MYLKP1* SNPs (MAFs >0.01) residing within the 4 kb *MYLKP1* promoter region, the core 1.4 kb of *MYLKP1* gene, and a 4 kb enhancer region were selected and genotyped in a colorectal cancer cohort. *MYLKP1* SNP influences on activity of *MYLKP1* promoter (2kb) was assessed by dual luciferase reporter assay.

**Results:**

Cancer cell lines, H441 and A549, exhibited increased *MYLKP1* expression, increased *MYLKP1* luciferase promoter activity, increased proliferation and migration. Genotyping studies identified two *MYLKP1* SNPs (rs12490683; rs12497343) that significantly increase risk of colon cancer in African Americans compared to African American controls. Rs12490683 and rs12497343 further increase *MYLKP1* promoter activity compared to the wild type *MYLKP1* promoter.

**Conclusion:**

*MYLKP1* is a cancer-promoting pseudogene whose genetic variants differentially enhance cancer risk in African American populations.

## Introduction

Pseudogenes are a type of long non-coding RNA originally derived from paralogues of functional genes. Historically, pseudogenes were considered non-functional genomic artifacts of catastrophic pathways, due to either the lack of regulatory elements or the presence of frameshift mutations [1]. However, nucleotides within these pseudogenes are conserved suggesting there is selective pressure to maintain the original genetic elements within the pseudogene [1]. Nearby regulatory elements regulate pseudogene transcription, and pseudogenes often share elements of the original gene's 5’ UTR and 3’ UTR regions allowing for differential regulation across tissue types. Recent evidence further suggests that pseudogenes may also serve as microRNA decoys leading to senescence susceptibility [2–4] and aberrantly regulate gene expression in cancer tissues [5–7]. For example, *PTENP1* [8] is a pseudogene of the tumor suppressor gene *PTEN* [9, 10] that is downregulated via methylation in renal cell carcinoma with *PTENP1* a competing non-endogenous RNA to suppress cancer progression [11]. Overall, pseudogenes require additional functional exploration in both cancer and non-neoplastic processes [5, 6].

We previously reported the functionality of *MYLKP1*, a pseudogene partially duplicated from *MYLK* on chromosome 3p13, with divergence from *MYLK* unique to higher hominids [12]. *MYLK* encodes three variants of myosin light chain kinase (MLCK) [13, 14] that participate in regulating cytoskeletal elements involved in maintaining cell integrity, contractility, motility, cell division [14, 15] and vascular barrier integrity [15, 16]. *MYLK* is associated with signaling pathways that include Rho/ROCK and Ca^2+^ signaling, which participate in colon cancer metastasis [17, 18]. *MYLK* downregulation is a hallmark of colon cancer metastasis, and *MYLK* mRNA and smooth muscle MLCK (smMLCK) protein are dysregulated in lung cancer [19, 20]. We previously demonstrated that genes influenced by *MYLK* expression are associated with a poor prognosis in a variety of cancer [21].

Evolutionarily, exons 13 through 17 of *MYLK* have been subjected to interchromosomal duplication, generating the partially duplicated *MYLKP1* pseudogene [22]. *MYLKP1* transcribes a sense strand of *MYLK* that decreases *MYLK* RNA stability [15]. Despite strong homology with the *smMLCK* promoter (~90%), the *MYLKP1* promoter is minimally active in normal bronchial epithelial cells but highly active as the *smMLCK* promoter in lung adenocarcinoma cells. Moreover, *MYLKP1* and *smMLCK* exhibit differential transcriptional profiling with *MYLKP1* strongly expressed in cancer cell lines (cervix, leukemia, uterus, colon) and tissues (colon, lymph node, vulva, bladder carcinoma), whereas *smMLCK* is highly expressed in non-neoplastic cells (bone marrow stem, uterine fibroblast, airway smooth muscle) and tissues (brain, breast, cervix, colon, liver, uterus, vein), tissues where *MYLKP1* expression is virtually absent. Thus, mechanistically, *MYLKP1* over-expression dramatically inhibits smMLCK expression in cancer cells and increases cell proliferation.

We have previously demonstrated that *MYLK* SNPs confer increased susceptibility to inflammatory disease that drives disease severity and mortality, particularly in African descent subjects with asthma and acute inflammatory lung injury [23, 24]. These results suggest the possibility that SNPs in the conserved *MYLKP1* promoter may exhibit higher minor allele frequencies (MAFs) in colon cancer subjects. Selected *MYLKP1* promoter SNPs were genotyped in a colorectal cancer cohort and further assessed by luciferase reporter promoter activity assays. Two known *MYLKP1* SNPs, rs12497343 (C>G) and rs12490683 (G>A) [25], affected *MYLKP1* promoter activity and were significantly associated with colon cancer risk in African Americans. These studies provide evidence for the functional involvement of *MYLKP1* pseudogenes in human carcinogenesis and suggest potential roles of *MYLKP1* as a novel population-specific diagnostic or therapeutic target in human colon cancer.

## Methods

### Primary cell cultures and cell lines

Beas-2b is a human bronchial epithelial cell line, H460 is a non-small cell lung cancer cell line, and A549 is an adenocarcinoma cell line provided by American Type Culture Collection (Manassa, VA, USA). All cell lines were grown according to the manufacturer’s protocol. Beas-2b and A549 were used to assess promoter function in *MYLKP1*. Promoter activity was measured using a standard luciferase assay that has been previously described [14, 15]. H23 non-small lung cancer cell-line, H441 adenocarcinoma, and H522 lung cancer were obtained from American Type Culture Collection (Manassa, VA, USA), were grown according to the manufacturer’s protocols, and were used to assess proliferation and migration.

### MYLKP1 luciferase assay

*MYLKP1* promoter (2kb) luciferase constructs were designed in a basic pGL4 vector containing each combination of the major and minor alleles of rs12497343 (C>G) and rs12490683 (G>A) (4 constructs in total). For dual luciferase reporter gene assays, cells grown in 12-well plates were cotransfected with 1 μg of the firefly luciferase vector containing the *MYLKP1* promoter and 20 ng of TK-renilla luciferase vector (Promega, Madison, WI, USA) using Fugene HD transfection reagent (Roche, Basel, Switzerland) as described previously [20].

### Cell proliferation and migration

For proliferation assays, cells were transfected with pcDNA 3.1 control or pcDNA 3.1 with *MYLKP1* gene clone using Fugene 6 transfection reagent (Roche) [15]. Two days after transfection, cells were selected with 400 μg/ml of Geneticin (G418; Sigma-Aldrich, St. Louis, MO, USA) and maintained with 200 μg/ml of G418. Cells grown in a 12-well plate with initial number of 10^5^ cells/well were harvested each day and counted using Countess Automated Cell Counter (Invitrogen, Carlsbad, CA, USA) up to 5 days.

### PCR differential detection

Total RNA was purchased from Agilent Technologies (Santa Clara, CA, USA) or isolated using TRIzol reagent (Invitrogen, Carlsbad, CA, USA) according to the manufacturer's protocol. For a conventional RT-PCR, each reaction was carried out with 2 μl cDNA, 0.5 μM forward (3bf) and reverse (3ar) primers, and 0.01 U Phusion DNA polymerase (Finnzymes, Espoo, Finland). Three-step PCR was performed according to the manufacturer's protocol as previously described [15]. The signal was detected by ethidium bromide staining after being run on a 2% agarose gel [15].

### Colorectal cases and controls

Individuals with colorectal cancer (n = 853; 400 AAs and 453 whites) who underwent surgical resection at the University of Chicago Department of Medicine between 1994 and 2008 were retrospectively ascertained from the Cancer Center and Pathology Department databases. Individuals known to have hereditary syndromes (familial adenomatous polyposis and Lynch syndrome) or inflammatory bowel disease were excluded. Available baseline characteristics including age, gender, race, colorectal tumor location, histological grade, depth of invasion, nodal involvement and recorded metastases.

Cancer-free control samples (n = 498; 302 AAs and 196 whites) were ascertained through our Pathology Department database (n = 305) and the University of Chicago Department of Medicine TRIDOM biobank (n = 93). The pathology-based controls included cancer-free individuals who had thyroidectomies and amputations and the biobank controls included cancer-free individuals visiting for a variety of bebign complaints. Controls were matched to cases by age at diagnosis, 10-year birth cohort, gender and race as recorded in the database. The details of sample collection and DNA preparation from archived surgical specimens have been validated and described previously [26].

### Genotyping

Using Tagger in Haploview, we selected a total of 14 SNPs with frequencies greater than 0.05 from the region spanning *MYLKP1*, the MLCK pseudogene. iPLEX assays for these 14 SNPs and 100 ancestry informative markers (AIMs) were designed using the Sequenom Assay Design software, and genotyped on the Sequenom MassARRAY platform. Selection and genotyping of the AIMs utilized have been published previously [27]. The methods for genotyping were also described previously [26].

### Genetic analysis

Utilizing AIMs information, the percent West African ancestry was estimated for each individual using STRUCTURE 2.1. Using prior population information from 60 Europeans and 131 West Africans, a model was run with K = 2 populations and a burn-in length of 30,000 iterations followed by 70,000 replications [28]. We excluded from the genetic analysis any African American subjects whose West African ancestry was < 0.25 (N = 10) and European American subjects whose West African ancestry was >0.1 (N = 46). Percent West African ancestry for heterozygotes and homozygotes was compared between controls and colorectal African American cases for each SNP genotyped. Percent West African ancestry was also compared via Welch two sample t-test for the homozygotes of the major allele and the homozygotes of the minor allele for controls and African American colorectal cancer cases. A p-value for false discovery rate (FDR) was performed using the Bejamini-Hochberg adjustment in R and reported with both unadjusted and adjusted p-values.

PLINK for utilized for the genetic analysis [29]. SNPs were tested for departures from Hardy-Weinberg equilibrium (HWE) which excluded three SNPs with p values < 0.005. We further removed any individual in which more than two SNPs were not successfully genotyped. After removal of poor quality DNAs, the average genotype rate in the remaining 11 SNPs was greater than 95%. We excluded SNPs with minor allele frequencies less than 0.05. Association with colorectal cancer was tested in European and African Americans separately. We tested association by calculation of the chi square statistic for the difference in allele frequency between cases and controls and calculated odds ratios and 95% confidence intervals. A p-value corrected for false discovery rate (FDR) was performed using the Benjamini-Hochberg adjustment in R for all tests (Chi-squared, dominance, recessive, and additive). We further tested dominant, recessive, and log-additive genetic models. Using logistic regression, p values were adjusted for West African ancestry estimates, sex, and age. Nominal significance was p< 0.05. Haplotype analysis was performed with the haplo.stats package in R. A chi-squared test was performed for each reported haplotype ([A,C], [A,G], [G,C], [G,G]) across African and European control and case haplotype frequencies.

## Results

### Detection of MYLKP1 expression in human cancer cells and transfected non-cancer cells

**Fig 1:**
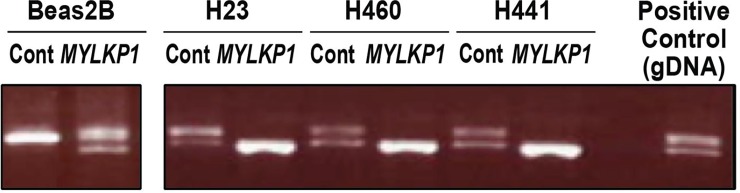
Simultaneous detection of MYLK and MYLKP via PCR in non-cancer cells (beas2b) and cancer cells (H23, H460, and H441).

*MYLKP1* contains a 72-base pair deletion compared with the *MYLK* gene (nt 342–413). PCR primers designed to flank the region containing the deletion were used to simultaneously amplify a segment of both *MYLK* and *MYLKP1* via traditional PCR techniques [15]. PCR products on a 2% agarose gel revealed two bands with the lower band reflecting the amplified *MYLKP1* mRNA transcript and the upper band reflecting *MYLK* mRNA transcript (Fig 1). We employed this method to detect *MYLKP*1 expression in several cell lines including human cancer cells (H23, H460, H441) and non-cancer epithelial cells (Beas2b) transfected with the *MYLKP1* plasmid (Fig 1). Genomic DNA (gDNA) showed both bands due to the presence of both amplicons in the human genome and was used as a positive control. Non-cancer lung epithelial cells (Beas2b) displayed expression of only *MYLK*, however, these cells expressed both *MYLKP1* and *MYLK* after transfection with *MYLKP1*. Cancer cells (H23, H460, H441) displayed basal expression of both *MYLK* and *MYLKP1*. After *MYLKP1* transfection, cancer cells preferentially over-express the smaller target, *MYLKP1*, indicating that *MYLKP1* suppresses expression of *MYLK* (Fig 1).

### MYLKP1 expression enhances cancer cell proliferation and migration

**Fig 2:**
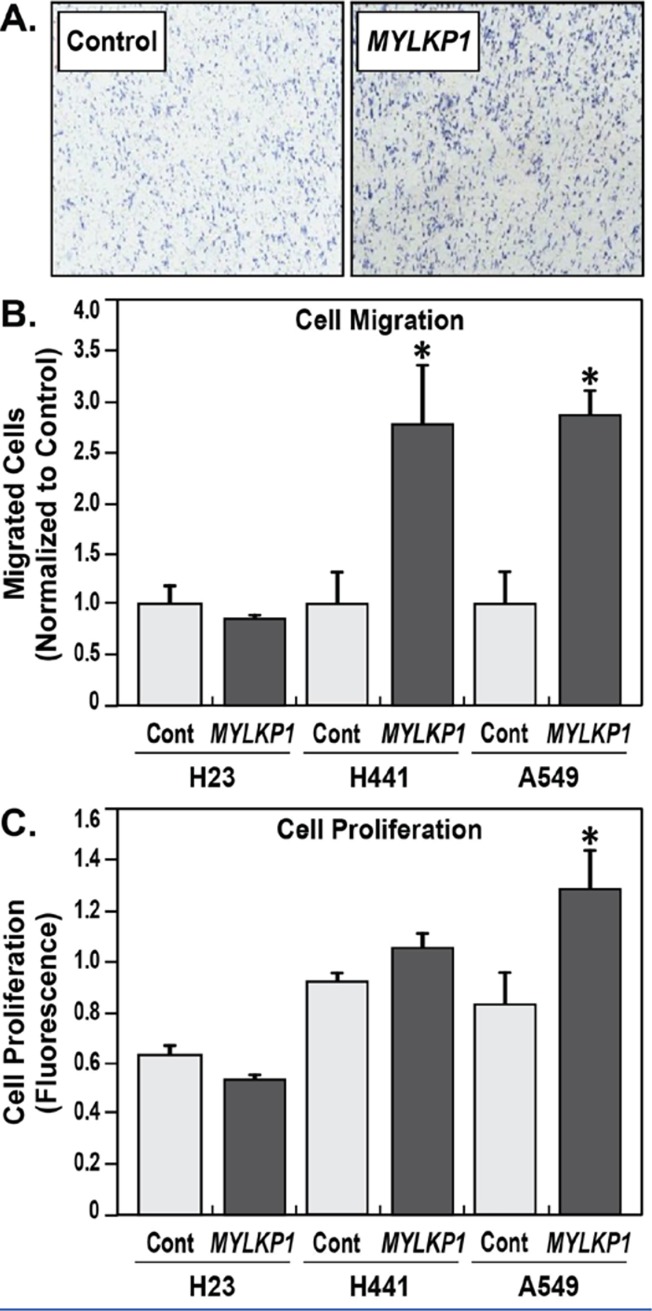
MYLKP increased cell proliferation and cell migration in lung cancer A549 and H441 cell lines. A. A549 and H441 cells transfected with *MYLKP* migrated more through a porous membrane significantly, compared to controls (*p<0.05). B. In A549 and H441 human lung adenocarcinoma cells, there was more proliferation in *MYLKP* transfected cells compared to controls (*p<0.05). C. MYLKP was transfected into lung cancer A549 and H441 cell lines, compared with cells transfected with empty vectors.

Histological staining demonstrated increased *MYLKP1* expression in A549 lung cancer cells (Fig 2A) corresponding with significant proliferation (Fig 2C) (p<0.05), consistent with our previous report that *MYLKP1* promotes proliferation in cancer cell lines and tissues [15]. Both H441 and A549 cell lines demonstrated significantly increased cell migration following *MYLKP1* transfection compared to control (p<0.05) (Fig 2B).

### MYLKP1 promoter SNPs increase colon cancer risk in african americans

**Fig 3:**
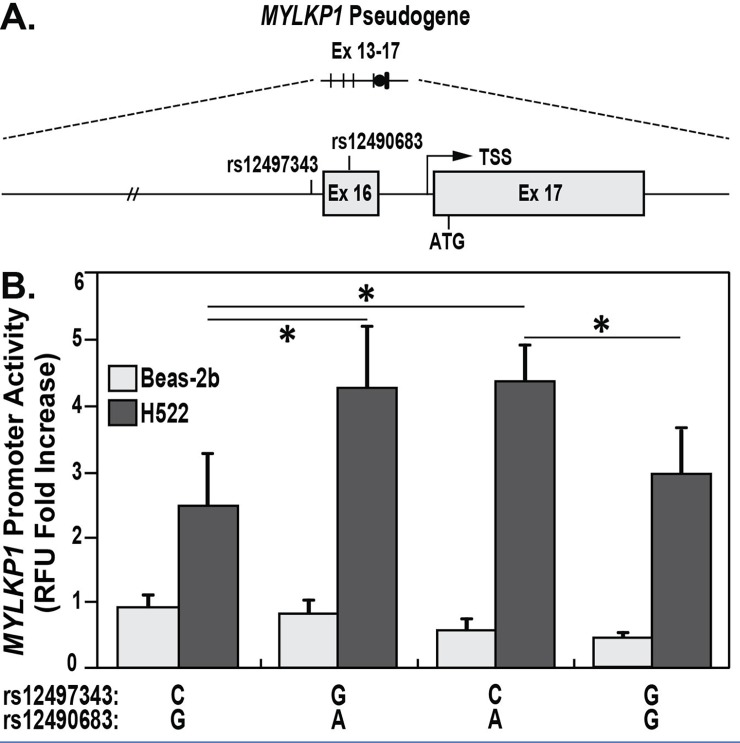
Genetic variants of M*YLKP* significantly increased *MYLKP* promoter activity in cancer cells. A. Two genetic variants of *MYLKP* rs12497343 and rs12490683 located in promoter region of *MYLKP* gene. B. In H522 cancer cells, *MYLKP* promoter activity was significantly increased compared to ones in non-cancer Beas-2b cells (*p<0.05). The haplotype G-A and C-A for two genetic variants of M*YLKP* rs12497343 C/G and rs12490683G/A was significantly increased *MYLKP* promoter activity in H522 cancer cells, compared to haplotype C-G and G-G (*p<0.05).

We have previously shown *MYLKP1* expression in cancer cell lines inhibits the expression of *MYLK* in cancer cells [15]. To further test *MYLKP1* as a potential oncogene, 11 *MYLKP1* SNPs surviving QC filtering were evaluated for genetic association in a cohort of African American and European American colorectal cancer subjects (Table 1). Only the *MYLKP1* SNP s12490683 achieved statistical significance in the analysis of European American colorectal cancer cases and controls. In the allele frequency test, both rs12497343 (p = 0.047) and rs12490683 (p = 0.023), present in the genomic region corresponding to the smooth muscle MLCK promoter in exon 16 and intron 15 (Fig 3A), were nominally associated with colorectal cancer risk in African Americans (Table 1). After adjustment for multiple testing (Benjamini and Hochberg false discovery rate—FDR), no SNP achieved significance (Table 1), however, these specific sites were selected for evaluation of potential functionality. We also tested dominant and recessive genetic models and found rs12497343 and rs12490683 achieved smaller p values in the recessive genetic model (0.018 and 0.002, respectively). After FDR correction, rs12490683 retained a p value < 0.05 (0 = 0.030) in the recessive genetic model (Table 1). Percent of West African heritage was compared between each SNP via chi-square test by genotype and corrected for FDR (Figure A in S1 File). By logistic regression, we tested a log-additive genetic model and adjusted for age, sex, and West African ancestry (Table 2). For rs7638312, a significance (p = 0.001) was reported for percentage of West African Ancestry between genotypes (Table 3), and rs7638312 was the only SNP with a significant difference in percentage West African Ancestry between genotypes (Table 3). After adjusting for age and sex, p values for rs12497343 and rs12490683 remained less than 0.05 but became insignificant after adjustment for West African ancestry (p values >0.05) (Table 2). A single SNP (rs4677496) in exon 17 region that we previously identified to be essential for smooth muscle *MYLK* expression [14] was excluded from the analysis due to a poor genotyping rate (Table 1). Haplotype analysis for rs12497343 and rs12490683 was performed for each haplotype ([A,C], [A,G], [G,C], [G,G]) across the four groups (European controls, European cases, African controls, and African cases), and chi-square p-values are reported with none being significant (Table A in S1 File).

**Table 1:** P values and odds ratios for associations with *MLCKP1* polymorphisms in African and European American colorectal cancer.

SNP	BP	MA	F_A	F_U	P_allele	OR (95% CI)	P_dom	P_rec	P_add	OR_add (95% CI)
African Americans										
rs10490780	75325508	G	0.140	0.148	0.687	0.94 (0.69,1.28)	0.761	0.694	0.698	0.94 (0.70,1.28)
rs9824516	75326959	A	0.132	0.124	0.657	1.08 (0.77,1.51)	0.575	0.845	0.663	1.08 (0.77,1.50)
rs7638312	75327606	C	0.061	0.057	0.775	1.07 (0.67,1.71)	NA	NA	0.780	1.07 (0.68,1.69)
rs6796799	75328126	A	0.273	0.270	0.907	1.02 (0.79,1.30)	0.919	0.636	0.907	1.02 (0.79,1.30)
rs4677497	75328974	G	0.155	0.164	0.666	0.93 (0.69,1.27)	0.581	0.853	0.665	0.93 (0.69,1.27)
rs12490683	75329934	A	0.238	0.186	0.023	1.37 (1.04,1.80)	0.208	**0.002**	0.029	1.35 (1.03,1.76)
rs12497343	75330074	G	0.264	0.216	0.047	1.30 (1.00,1.69)	0.159	**0.018**	0.041	1.33 (1.01,1.74)
rs6801219	75332618	G	0.102	0.121	0.293	0.83 (0.59,1.18)	0.282	0.802	0.312	0.84 (0.60,1,12)
rs2091870	75333283	G	0.338	0.345	0.781	0.97 (0.77,1.22)	0.509	0.665	0.784	0.97 (0.77,1.22)
rs4552385	75336163	C	0.466	0.457	0.747	1.04 (0.83,1.29)	0.711	0.342	0.753	1.04 (0.83,1.28)
rs4677503	75338306	A	0.323	0.311	0.466	1.09 (0.86,1.39)	0.357	0.946	0.482	1.09 (0.86,1.36)
European Americans										
rs6796799	75328126	A	0.134	0.124	0.623	1.10 (0.75,1.61)	NA	NA	0.62	1.10 (0.75,1.61)
rs4677497	75328974	G	0.118	0.112	0.795	1.06 (0.70,1.59)	NA	NA	0.80	1.05 (0.71,1.58)
rs12490683	75329934	G	0.381	0.364	0.603	1.07 (0.83,1.39)	0.831	0.432	0.58	1.08 (0.82,1.41)
rs12497343	75330074	C	0.393	0.382	0.714	1.05 (0.81,1.36)	0.609	0.964	0.69	1.06 (0.80,1.39)
rs2091870	75333283	A	0.368	0.339	0.374	1.13 (0.87,1.48)	0.728	0.165	0.35	1.14 (0.87,1.50)
rs4552385	75336163	T	0.305	0.300	0.882	1.02 (0.77,1.35)	0.724	0.304	0.88	1.02 (0.77,1.37)
rs4677503	75338306	G	0.333	0.311	0.470	1.11 (0.84,1.45)	0.963	0.109	0.47	1.10 (0.84,1.44)

P_allele is the p value obtained in the chi square test of allele frequency, and it is associated with odds ratio (OR) and 95% confidence interval (CI) in the adjacent column; P_dom is the value obtained assuming a dominant genetic model; P_rec is the value obtained assuming a recessive genetic model. P_add is the unadjusted p value obtained from a logistic regression assuming a log-additive genetic model, and it is associated with OR and 95% CI in the adjacent column. NA indicates that the frequency of one of the genotypes was too low to perform the test of the model. For the nominally significant SNPs, rs12490683 and rs12497343, the lowest p values obtained in the analysis are in **bold.** There are four fewer SNPs displayed in the European American part of the table because the minor allele frequency of the excluded SNPs was less than 0.05.

BP, base pair position on chromosome 3, GRCh38; F_A, frequency of minor allele in colorectal cancer cases; F_U, frequency of minor allele in controls; MA, minor allele; SNP, single nucleotide polymorphism.

**Table 2:** P values and odds ratios for associations with *MYLKP1* polymorphisms in African and European American colorectal cancer, adjusted for age, sex, and West African ancestry.

SNP	BP	MA	P_adj1	OR_adj1	P_adj2	OR_adj2
African Americans						
rs10490780	75325508	G	0.791	0.96	0.718	0.95
rs9824516	75326959	A	0.452	1.14	0.518	1.12
rs7638312	75327606	C	0.623	1.12	0.672	1.11
rs6796799	75328126	A	0.728	1.05	0.811	1.03
rs4677497	75328974	G	0.630	0.93	0.603	0.92
**rs12490683**	**75329934**	**A**	**0.138**	**1.24**	**0.038**	**1.33**
**rs12497343**	**75330074**	**G**	**0.220**	**1.19**	**0.078**	**1.28**
rs6801219	75332618	G	0.383	0.86	0.318	0.84
rs2091870	75333283	G	0.421	0.91	0.744	0.96
rs4552385	75336163	C	0.842	1.02	0.660	1.05
rs4677503	75338306	A	0.545	1.08	0.367	1.11
European Americans						
rs6796799	75328126	A	0.610	1.11	0.558	1.12
rs4677497	75328974	G	0.809	1.05	0.781	1.06
rs12490683	75329934	G	0.601	1.08	0.579	1.08
rs12497343	75330074	C	0.673	1.06	0.685	1.06
rs2091870	75333283	A	0.354	1.14	0.347	1.14
rs4552385	75336163	T	0.869	1.03	0.915	1.02
rs4677503	75338306	G	0.496	1.10	0.494	1.10

P_adj1 is the p value for association adjusted for age, sex, and West African ancestry and its associated odds ratio (OR_adj1) is in the adjacent column. P_adj2 is the p value for association adjusted for age and sex and its associated OR (OR_adj2) is in the adjacent column. There are four fewer SNPs displayed in the European American part of the table because the minor allele frequency of the excluded SNPs was less than 0.05.

BP, base pair position on chromosome 3, GRCh38; MA, minor allele; SNP, single nucleotide polymorphism. Bolded are the two SNPs chosen for functional analyses

**Table 3:** Percentage of West African Heritage by SNP for African American Colorectal Cancer Cases.

SNP	Minor Allele	West African Ancestry	P-Value (Adjusted)^a^
rs10490780	G	0.838	0.926 (0.926)
rs9824516	A	0.826	0.186 (0.292)
rs7638312	C	0.826	0.001 (0.011)
rs6796799	A	0.815	0.596 (0.755)
rs4677497	G	0.809	0.821 (0.903)
rs12490683	A	0.784	0.068 (0.193)
rs12497343	G	0.788	0.040 (0.193)
rs6801219	G	0.836	0.154 (0.282)
rs2091870	G	0.807	0.136 (0.282)
rs4552385	C	0.817	0.618 (0.755)
rs4677503	A	0.804	0.070 (0.193)

West African Ancestry heritage for each SNP. FDR is calculated using the Benjamini and Hochberg adjusted p-values via the R program p.adjust.

a Chi-squared p value calculated for each SNP.

### MYLKP1 SNPs associated with colon cancer risk alter MYLKP1 promoter activity

After confirming the role of *MYLKP1* in the H441 and A549 cell lines (Fig 2), we investigated the role of two SNPs of interest, rs12490683 G>A and rs12497343 C>G, in regulation of *MYLKP1* promoter activity (Fig 3B). *MYLKP1* promoter luciferase reporter assays were conducted in a human adenocarcinoma cell line (H522) and a non-cancer cell line (Beas2b). The wild type vector, utilizing the major allelic pairing (rs12490683-G and rs12497343-C) showed *MYLKP1* to be significantly upregulated in cancer cells (H522) over epithelial cells (Beas-2b) (p<0.05) (Fig 3B). Furthermore, transfection of a *MYLKP1* promoter luciferase reporter harboring the minor allelic pairing (rs12497343-G and rs12490683-A) into H522 cancer cells resulted in significantly greater promoter activity (p<0.05) when compared to the major allelic pairing in H522 cancer cells (Fig 3B).

## Discussion

We and others have demonstrated that the pseudogene, *MYLKP1*, located on 3p12.3 (HGNC ID:7591) representing an intrachromosomal duplication of exons 13 to 17 of *MYLK* copied from 3q21.1 (HGNC ID:7590) [19, 30], is selectively expressed in cancer, regulates MLCK levels, and increases cancer cell proliferation *in vitro* [15, 22]. While *MYLKP1* and functional *MYLK* share high levels of DNA sequence similarity (93%), *MYLK* is an intricate gene spanning over 270 kb and containing 34 exons which via alternative splicing [2], generates 9 transcripts that encode 3 proteins including a 220 kDa non-muscle MLCK isoform (nmMLCK), a 130 kDa smooth muscle MLCK isoform (smMLCK) [20], and a 20 kDa protein isoform known as telokin [31]. *MYLK* encodes the multi-functional myosin light chain kinase (MLCK) which is involved in diverse functions in multiple types of cancer.

Similar to other documented pseudogenes [30, 32, 33], we have shown that *MYLK* and *MYLKP1* have a pseudogene/parent gene crosstalk relationship. Due to high sequence similarity to the functional gene, pseudogenes often pose a challenge for gene prediction programs with frequent misidentification as real genes. For instance, initial interpretation of the sequence data from human chromosome 22 indicated that 19% of the coding sequences are pseudogenic [12]. More robust direct surveys of pseudogenes revealed that the estimated number of pseudogenes is ~20,000 [6, 14], a figure comparable to the number of protein-coding genes in the human genome [17]. Despite the abundance of pseudogenes in the human genome, the pathophysiological roles of pseudogenes remain poorly understood. Unlike duplicated pseudogenes and retrotransposed pseudogenes [14, 15], other pseudogenes are potentially transcriptionally active, expressing mRNAs utilizing their own promoters or adjacent promoters [16, 18]. Duplicated pseudogenes including *MYLKP1*, generated by tandem duplication or unequal crossover events [34], produce antisense RNAs and inhibit functional gene expression through antisense-sense mechanism [8] with functional effects on human disease [5, 15, 35, 36].

We identified *MYLKP1* as a pseudogene of *MYLK* that regulates levels of cellular MLCK and is selectively expressed in cancer cells, a finding observed with other pseudogenes [5, 37, 38]. The pseudogene, *PTENP1*, acts as a microRNA decoy and thus helps maintain cellular levels of *PTEN*, however, the *PTENP1* locus is selectively lost in specific cancer cells, resulting in decreased *PTEN* expression and increased proliferation [5]. Our studies indicate that *MYLKP1* may function similarly to regulate levels of MLCK, a Ca^2+^/CaM-dependent enzyme that functions as a critical regulator of cytoskeletal function [39], cell contraction, cytokinesis [10], cellular motility [11, 40–42], mitosis [7], apoptosis [32], cell migration [31, 39] and inflammatory cell trafficking [33]. Both the smMLCK and nmMLCK isoforms are essential participants in many key pathophysiologic features of human diseases including essential hypertension [4, 20, 22], acute inflammatory lung injury, asthma [14, 43] as well as breast, pancreatic and non-small cell lung cancer [44, 45]. *MYLK* expression is also increased in angiogenesis and in tumors that exhibit increased invasiveness [1]. We have previously shown that nmMLCK is an independent predictor of poor clinical outcome among cancer patients that was independent of other clinic-pathologic factors [2]. Specifically, MLCK participates in migration, metastasis, and increased cellular proliferation [6, 46–48].

Previously, we have shown that an upregulation in *MYLKP1* mRNA expression produces a functional transcript in multiple cancer cell lines [14, 15], and this corresponds with the downregulation of functional *MYLK* mRNA in cancer cell lines. *MYLKP1* expression inhibits the functional gene products of *MYLK*, including smMLCK protein expression. We attempted to elucidate a potentially active biological role for *MYLKP1* and to clarify its participation as a candidate gene in cancer risk. We now show that *MYLKP1* selectively transcribes mRNA in cancer cells and dramatically decreases the expression of the functional *MYLK* (Fig 1). Moreover, expression of the pseudogene increases cell proliferation of normal and cancer cells (Fig 2A, Fig 2B), indicating an active role of *MYLKP1* during carcinogenesis. We previously demonstrated that *MYLKP1* is selectively expressed in cancer cells, functions as a regulator of MLCK levels, and increases cancer cell proliferation *in vitro* [14]. The potential for cross-talk between the parent gene and the pseudogene (*MYLK* and *MYLKP1*) and nmMLCK's potential as a cancer biomarker provide unique targets for cancer therapeutics that have the potential to affect cancer cell proliferation.

The rate of colon cancer mortality among African Americans is significantly higher than Caucasian Americans independent of socioeconomic status [49]. Mutations with a higher MAF in African Americans with colon cancer could provide a particularly valuable therapeutic target, and the unique regulation of the parent gene (*MYLK*) by its pseudogene (*MYLKP1*) provides a possible mechanistic explanation for the increased severity of colon cancer and its development at younger ages in African Americans [49]. Two promoter SNPs (rs12497343 and rs12490683) in the *MYLKP1* promoter region are promising candidates that could contribute to the regulation of *MYLKP1* in cancer. These SNPs were discovered to be significant among populations of African descent and could contribute to health disparity in colon cancer outcomes but require independent replication for confirmation of this potentially important association. Improved reference panels that account for the unique diversity in African American genetic backgrounds and use of imputation to overcome obstacles with the homology between the *MYLK* and *MYLKP1* promoter regions, may reveal unique therapeutic targets for cancer and elucidate mechanisms and pathways that contribute to greater colon cancer severity in African American populations [50]. Either next generation sequencing or imputation of the *MYLKP1* promoter could provide genotypes for the rs4677496 SNP, which was unable to be genotyped.

Together, these studies, which provide further support for the functional involvement of pseudogenes in human pathobiology, suggest *MYLKP1* should be considered as a novel diagnostic or therapeutic target in human cancer.

## Supporting information

S1 File A file containing supplementary information **(Table A)** Haplotype frequencies for African American and European American controls and cases were calculated via haplo.stats package in R. A chi-squared test between groups were performed per haplotype, and raw p-values were reported. (**Figure A)** A histogram of West African ancestry was plotted in R. The plot includes both the ratio of West African ancestry in both African American colorectal cancer patients and controls.
(PDF)

S1 Dataset Supplementary data on the genotyping of both colorectal cancer patients and controls provided for open access. (ZIP)

## Abbreviations

MLCK: myosin light chain kinase
MYLK: myosin light chain kinase
smMLCK: smooth muscle MLCK
MLKP1: myosin light chain kinase pseudo gene
