# Supplementary material for: Single nucleotide polymorphisms in the MYLKP1 pseudogene are associated with increased colon cancer risk in African Americans
Source: PLoS One. 2018 Aug 30;13(8):e0200916. doi: 10.1371/journal.pone.0200916 (PMC6116948; doi:10.1371/journal.pone.0200916)

Supplementary Table A: Haplotypes for rs12490683 and rs12497343 combined and by disease status and ethnicity

rs12490683	rs12497343	Combined Frequency	AA Control	AA Case	EA Control	EA Case	Chi Squared (All groups)	P value
[G]	[C]	0.68045	0.76151	0.73632	0.61675	0.60635	0.0283	0.9987
[G]	[G]	0.03080	0.05099	0.04602	0.01523	0.01086	0.0416	0.9978
[A]	[C]	0.00339	0	0	0	0.00978	0.0293	0.9987
[A]	[G]	0.28535	0.18750	0.21766	0.36802	0.37301	0.1000	0.9918

Supplementary Figure A: Histogram of ratio of West African Ancestry in African American controls and African American Colorectal Cancer Patients

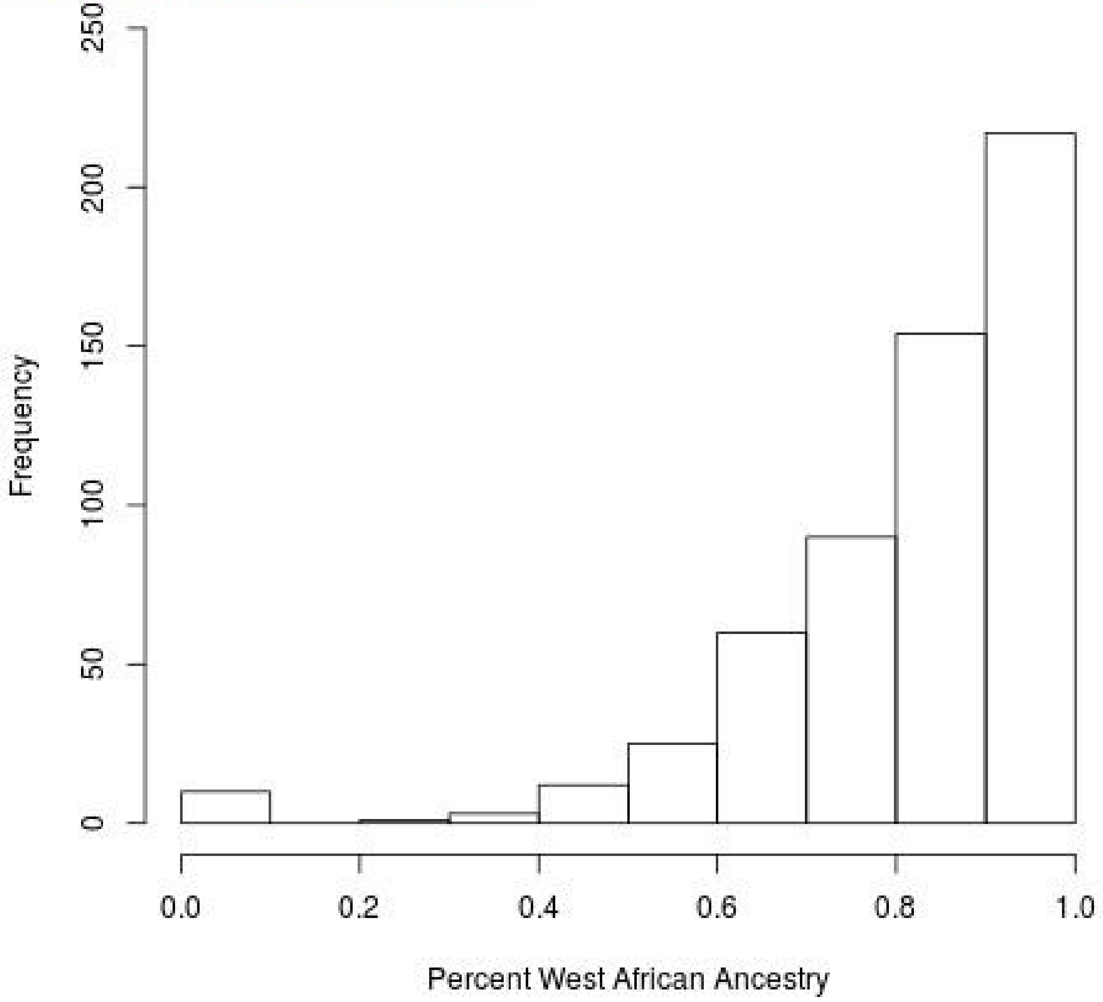

Supplement: S1 File — A file containing supplementary information (Table A) Haplotype frequencies for African American and European American controls and cases were calculated via haplo.stats package in R. A chi-squared test between groups were performed per haplotype, and raw p-values were reported. (Figure A) A histogram of West African ancestry was plotted in R. The plot includes both the ratio of West African ancestry in both African American colorectal cancer patients and controls. (PDF) [file pone.0200916.s001.pdf]
